# Chicago Hotel for Invalids

**Published:** 1868-08-01

**Authors:** 


### *Chicago Hotel for Invalids.*

Attention is directed to the advertisement of the reorganization of this much needed institution. It will be noticed that patients have full liberty to select their own medical atttendants, provided these are of recognized respectability. With this modification, and the other advantages afforded, we have every confidence that the Hotel for Invalids will soon acquire general popularity with both the profession and the public. From personal knowledge of the intentions of the managers, we are sure that everything will be conducted with scrupulous regard for the proprieties of the code.

